# Urinary Concentrations of Parabens and Other Antimicrobial Chemicals and Their Association with Couples’ Fecundity

**DOI:** 10.1289/EHP189

**Published:** 2016-06-10

**Authors:** Melissa M. Smarr, Rajeshwari Sundaram, Masato Honda, Kurunthachalam Kannan, Germaine M. Buck Louis

**Affiliations:** 1 Office of the Director, and; 2 Biostatistics and Bioinformatics Branch, Division of Intramural Population Health Research, *Eunice Kennedy Shriver* National Institute of Child Health and Human Development, National Institutes of Health, Department of Health and Human Services, Rockville, Maryland, USA; 3 Wadsworth Center, New York State Department of Health, Albany, New York, USA; 4 Department of Environmental Health Sciences, School of Public Health, State University of New York at Albany, Albany, New York, USA

## Abstract

**Background:**

Human exposure to parabens and other antimicrobial chemicals is continual and pervasive. The hormone-disrupting properties of these environmental chemicals may adversely affect human reproduction.

**Objective:**

We aimed to prospectively assess couples’ urinary concentrations of antimicrobial chemicals in the context of fecundity, measured as time to pregnancy (TTP).

**Methods:**

In a prospective cohort of 501 couples, we examined preconception urinary chemical concentrations of parabens, triclosan and triclorcarban in relation to TTP; chemical concentrations were modeled both continuously and in quartiles. Cox’s proportional odds models for discrete survival time were used to estimate fecundability odds ratios (FORs) and 95% confidence intervals (CIs) adjusting for *a priori*–defined confounders. In light of TTP being a couple-dependent outcome, both partner and couple-based exposure models were analyzed. In all models, FOR estimates < 1.0 denote diminished fecundity (longer TTP).

**Results:**

Overall, 347 (69%) couples became pregnant. The highest quartile of female urinary methyl paraben (MP) concentrations relative to the lowest reflected a 34% reduction in fecundity (aFOR = 0.66; 95% CI: 0.45, 0.97) and remained so when accounting for couples’ concentrations (aFOR = 0.63; 95% CI: 0.41, 0.96). Similar associations were observed between ethyl paraben (EP) and couple fecundity for both partner and couple-based models (*p*-trend = 0.02 and *p*-trend = 0.05, respectively). No associations were observed with couple fecundity when chemicals were modeled continuously.

**Conclusions:**

Higher quartiles of preconception urinary concentrations of MP and EP among female partners were associated with reduced couple fecundity in partner-specific and couple-based exposure models.

**Citation:**

Smarr MM, Sundaram R, Honda M, Kannan K, Buck Louis GM. 2016. Urinary concentrations of parabens and other antimicrobial chemicals and their association with couples’ fecundity. Environ Health Perspect 124:730–736; http://dx.doi.org/10.1289/EHP189

## Introduction

Antimicrobial agents destroy or inhibit the growth and spread of microorganisms such as bacteria and fungi. Human exposure to antimicrobial agents can be attributed to the use of personal care and food products (Considine 2005; Sung et al. 2013). Specifically, parabens are a class of nonpersistent endocrine-disrupting chemicals (EDCs) with antimicrobial properties, found in a variety of commercial products including pharmaceuticals, nutritional supplements, personal care products, and food items (Dodge et al. 2015a; Guo and Kannan 2013; Liao et al. 2013; Liao and Kannan 2014; Schettler 2006). Additionally, the antibacterial agents triclosan (TCS) and triclocarban (TCC) have widespread commercial application as additives in personal care products, textiles, and plastic kitchenware (Food and Drug Administration 2016; Gao and Cranston 2008; Liao and Kannan 2014). The growing use of just antibacterial household products is evident in the increase from a few dozen to > 700 consumer products on the market; sales in the United States alone are estimated to reach 1.6 billion dollars by the year 2017 (Levy 2001; Smith 2013; Tan et al. 2002). Specific to personal care products, the assessment of 395 soaps sold in a national chain store and regional grocery store in the United States found antibacterial agents to be present in 76% of liquid soaps and 29% of bar soaps (Perencevich et al. 2001).

Biomonitoring data underscore the ubiquitous prevalence of these environmental chemicals, with 99%, 92%, and 75% of the U.S. general population having detectable concentrations of methyl paraben (MP), propyl paraben (PP), and TCS, respectively, in urine (Calafat et al. 2010). These data also reported gender differences in estimated paraben exposure but not TCS; urinary MP and PP concentrations in females were higher than those found in males. Parabens, having weak estrogenic properties in comparison with estradiol, possess an affinity for the estrogen receptor in a manner directly related with the size of the alkyl group on the paraben (Witorsch and Thomas 2010). The reproductive toxicity of these antimicrobial chemicals has been demonstrated primarily in rodent models. In one study, daily subcutaneous exposure to parabens among 55 female neonatal rats resulted in abnormalities in ovarian folliculogenesis—increased primordial follicles and decreased early primary follicles (Ahn et al. 2012). Another study of oral exposure to parabens among prepubertal female rats found decreased ovarian weight and histopathological abnormalities in the ovaries, among other adverse effects (Vo et al. 2010). On the male side, rats age 19–21 days exposed to butyl paraben (BP) or PP via diet for 8 weeks were found to have reduced secretion of testosterone and sperm production (Oishi 2001, 2002). Albeit scarce, epidemiological findings suggest associations between paraben exposure and ovarian aging, sperm DNA damage, and reduced fertility (Dodge et al. 2015b; Meeker et al. 2011; Smith et al. 2013). Nevertheless, studies of preconception urinary levels of antimicrobial chemicals (i.e., parabens, TCS, and TCC) and prospectively assessed couple fecundity, as measured by time to pregnancy (TTP) or the number of menstrual cycles required to achieve pregnancy, are lacking, and therefore serve as the motivation for this study.

## Materials and Methods

### Study Population

The Longitudinal Investigation of Fertility and the Environment (LIFE) study population comprised 501 reproductive-age couples who were recruited from 16 counties in Michigan and Texas between 2005 and 2009. Couples were recruited upon discontinuing contraception for the purpose of becoming pregnant, as previously described (Buck Louis et al. 2011). Inclusion criteria were minimal: females 18–40 years of age and males ≥ 18 years; couples in a committed relationship and planning to try for pregnancy or currently off contraception for ≤ 2 months; females with menstrual cycles between 21 and 42 days without any injectable hormonal contraceptives in the past year; and an ability to communicate in English or Spanish. Institutional review board approvals were obtained from all collaborating institutions; couples gave written informed consent before study participation and any data collection.

### Biospecimen Collection and Analysis

During the enrollment home visit, each partner of the couple provided a spot urine and nonfasting blood sample. Using established protocols (Asimakopoulos et al. 2014), MP, EP, PP, BP, benzyl paraben (BzP), heptyl paraben (HP), 4-hydroxy benzoic acid (4-HB), 3,4-dihydroxy benzoic acid (3,4-DHB), methyl-protocatechuic acid (OH-MeP), and ethyl-protocatechuic acid (OH-EtP) were quantified along with TCS and TCC by the Wadsworth Center, New York State Department of Health. Specifically, 300 μL of 1 M ammonium acetate containing 30 U of β-glucuronidase (pH = 5.5) was added to 500 μL of urine sample, followed by incubation at 37°C for 12 hr. Target analytes were extracted three times with ethyl acetate and were quantified as nanograms/milliliter by ultra-performance liquid chromatography (Acquity I Class; Waters, Milford, MA) coupled with an electrospray triple quadrupole tandem mass spectrometry (UPLC-ESI-MS/MS) (API 5500; AB SCIEX, Framingham, MA); separation of target analytes was carried by a Kinetex C18 (1.3 u, 100A, 50 × 2.1 mm) column (Phenomenex; Torrance, CA) with a SecurityGuard® guard column (Phenomenex; Torrance, CA). Quality assurance and quality control parameters included procedural blanks, matrix spikes, and duplicate analysis of samples. Labeled internal standards were spiked into all samples and quantification was by isotope dilution. Creatinine was quantified using a Roche/Hitachi Model 912 clinical analyzer (Dallas, TX) and the Creatinine Plus Assay. In addition, nonfasting blood samples were collected from each partner to measure serum cotinine using liquid chromatograph–isotope dilution tandem mass spectrometry and as reported in ng/mL (Bernert et al. 1997).

### Assessment of Lifestyle and Couple Fecundity

To capture couples’ lifestyles and reproductive health histories, interviews were conducted followed by standard anthropometric assessments to measure body mass index (BMI) (Lohman et al. 1988). Couples completed daily journals about lifestyle and women recorded menstruation and pregnancy results. Female partners were instructed in the use of the Clearblue® Easy home fertility monitor, which tracks esterone-3-glucuronide (E_3_G) and luteinizing hormone (LH), allowing couples to time intercourse relative to peak fertility, which is a proxy marker of ovulation (Behre et al. 2000). Last, female partners used the Clearblue® Digital home pregnancy test, capable of detecting 25 mIU/mL human chorionic gonadotropin (hCG), to test for pregnancy on the day of expected menses (Johnson et al. 2015). Therefore, it was possible to differentiate between couples achieving pregnancy in the first few weeks postenrollment (TTP = 0 completed cycles) and those achieving pregnancy during the first fully observed menstrual cycle (TTP = 1). Couples were followed up to 12 months of trying at which point TTP was censored.

### Statistical Analysis

Univariate analyses were performed to assess all chemical distributions and relevant covariates. Female and male partners’ lifestyle characteristics were compared using independent *t*-tests and chi-square tests for continuous and categorical covariates, respectively. Urinary creatinine and chemical concentrations were natural-log transformed (x + 1) to normalize distributions. The correlation between male and female partner’s chemical concentrations was examined with the use of Spearman rank analyses. Median and accompanying interquartile ranges (IQRs) of preconception urinary chemical concentrations were calculated; medians were compared between female and male partners using Wilcoxon–Mann–Whitney test. To avoid biasing point estimates when assessing health outcomes, the unadjusted ln-transformed instrument measured values for all chemicals were used in statistical models and ln-transformed urinary creatinine was included as a covariate (Lubin et al. 2004; Richardson and Ciampi 2003; Schisterman et al. 2006). HP was not detected in any urine samples. For BzP and TCC, > 80% of urine samples had concentrations below the limit of quantification (LOQ) prompting us to model them as above/below LOQ. These chemicals were excluded from continuous linear models; point estimates and *p*-values for dichotomous variables are presented for models of dichotomous urine concentrations. Missing concentrations of urinary chemicals for females (6%) and males (12%) stemming from insufficient urine volume for chemical quantification (due to previous use of samples) and missing covariate data (2%) were imputed using Markov chain Monte Carlo methods (Schafer 1997) to minimize bias (Desai et al. 2011; White and Carlin 2010). Imputations were performed under the assumption that missing urine samples were not dependent upon couples’ TTP and were therefore assumed to be missing at random. Box-Cox transformation was performed on imputed values to achieve normality in the highly skewed imputed values (Osborne 2010). We defined statistical significance as a two-sided *p*-value < 0.05.

Cox proportional odds models for discrete survival time and allowing for a cycle-varying intercept were used to estimate fecundability odds ratios (FORs) and 95% confidence intervals (CIs) (Cox 1972). We also accounted for left truncation or time couples were off contraception at enrollment, and right censoring due to attrition. FORs estimate the odds of pregnancy each cycle conditional on not being pregnant in the previous cycle per unit of chemical change. FOR estimates < 1.0 reflect diminished fecundity (longer TTP), and FORs > 1 reflect enhanced fecundity (shorter TTP).

We first modeled each partner’s chemical concentrations and then jointly modeled both partners’ concentrations in keeping with the couple-dependent nature of human reproduction. We *a priori* defined confounders as age (years), BMI (kg/m^2^) categorized as [normal (< 25), overweight (25–30), obese (30–35) and morbidly obese (> 35)], ln(creatinine) (mg/dL), active preconception smoking [serum cotinine above/below 10 ng/mL (Benowitz et al. 2009; Hukkanen et al. 2005)], race/ethnicity (nonwhite/white), and household income (above/below $70,000) (Augood et al. 1998; Calafat et al. 2008, 2010; Curtin et al. 2015; Dunson et al. 2002; Hassan and Killick 2004; Martin et al. 2015; Ramlau-Hansen et al. 2007; Smith et al. 2013).

Fecundity models were first run with continuous urinary concentrations to assess potential linear associations between urinary chemicals and fecundity then in quartiles to assess nonlinear relationships. Wald tests were performed to test for linear trend across quartiles of chemical concentrations.

In light of the exploratory nature of this analysis, we undertook sensitivity analysis to assess the robustness of our findings. First, we repeated the above analyses restricting to couples without imputed chemical data, to assess introduction of bias by imputation methods. Next, to examine the role of time with respect to urinary chemical assessment and TTP, models included in the primary analysis were performed restricting the data to include couples with a TTP < 2 cycles. All analyses were performed using SAS software (version 9.4; SAS Institute Inc., Cary, NC).

## Results

Among the 501 enrolled couples, 347 (69%) had an observed hCG-confirmed pregnancy. The women and men in LIFE Study were primarily college educated (95% and 91%, respectively) and predominantly of non-Hispanic white ethnicity and race (79%). The mean female and male ages were 30.0 ± 4.1 and 31.8 ± 4.9 years, respectively (Table 1). The prevalence of cigarette smoking before conception was lower among females than male partners (12% and 22%, respectively).

**Table 1:** Comparison of partners by select baseline characteristics, the LIFE Study (*n* = 501).

Characteristics	Females [*n* (%)]	Males [*n* (%)]
Age (years) at baseline^a^*	30.0 ± 4.1	31.8 ± 4.9
BMI at baseline*
Under/healthy (BMI < 25)	229 (46)	84 (17)
Overweight (25 ≤ BMI < 30)	136 (27)	206 (42)
Obese (30 ≤ BMI < 35)	66 (13)	131 (26)
Morbidly obese (BMI ≥ 35)	69 (14)	75 (15)
Missing values	1 (< 1)	5 (< 1)
Mean ± SD*	27.6 ± 7.3	29.8 ± 5.6
Race/Ethnicity
Non-Hispanic white	393 (79)	394 (79)
Non-Hispanic black	24 (5)	23 (5)
Hispanic	50 (10)	45 (9)
Other	31 (6)	36 (7)
Missing values	3 (< 1)	3 (< 1)
Household income
< $70,000	157 (32)	163 (33)
≥ $70,000	334 (68)	328 (67)
Missing values	10 (2)	10 (2)
Parity conditional on gravidity
No prior pregnancy	210 (42)	215 (43)
Prior pregnancy
Without live birth(s)	53 (11)	44 (9)
With live birth(s)	235 (47)	239 (48)
Missing values	3 (< 1)	3 (< 1)
Active smoking status*
Yes (serum cotinine ≥ 10 ng/mL)	58 (12)	106 (22)
No (serum cotinine < 10 ng/mL)	431 (88)	387 (79)
Missing values	12 (2)	9 (2)
BMI, body mass index (kg/m^2^). ^***a***^Mean ± SD. *p < 0.05 from independent *t*-test for continuous characteristics or chi-square test for categorical characteristics.		

The median and accompanying IQRs for creatinine-adjusted urinary concentrations of parabens and their metabolites and the antibacterial agents TCS and TCC for each partner are displayed in Table 2. Generally, parabens and antibacterial chemical concentrations were readily detectable in all couples, ranging from 78% to 100% of concentrations > LOQ in women and 74% to 100% in males. TCS was detected in 93% and 87% of females and males, respectively. Creatinine-adjusted urinary concentrations of most parabens presented in Table 2 were significantly higher for females than males (*p* < 0.0001); unadjusted chemical concentrations were similar (see Table S1).

**Table 2:** Distribution of urinary creatinine-adjusted antimicrobial phenolic concentrations by partner.

Chemical (μg/g)	LOQ	Female (*n* = 470)		Males (*n* = 439)		Partners’ chemical correlation
		% < LOQ^*a*^	Median (IQR)	% < LOQ^*a*^	Median (IQR)	
Creatinine**	3.5	0	77.9 (35.1–136)	0	140 (72.3–201)	0.19**
MP**	0.2	0	60.0 (17.8–154)	1	6.37 (1.84–25.8)	0.06
EP**	0.1	5	1.51 (0.457–8.97)	18	0.33 (0.13–1.25)	0.08
PP**	0.05	2	19.5 (5.45–59.7)	5	1.48 (0.426–5.73)	0.02
BP**	0.1	22	0.83 (0.12–4.26)	70	0.03 (0.01–0.16)	0.01
BzP*	0.1	84	0.02 (0.00–0.06)	91	0.01 (0.00–0.04)	0.35**
HP	0.1	100	0.00 (0.00–0.00)	100	0.00 (0.00–0.00)	0.04
4-HB**	2	0	671 (424–1,138)	0	478 (335–692)	0.15*
3,4-DHB**	5	1	43.2 (27.7–73.0)	1	26.1 (16.8–45.9)	0.15*
OH-MeP**	1	0	31.4 (14.3–65.9)	1	18.3 (9.14–41.7)	0.23**
OH-EtP**	1	15	6.46 (1.87–20.7)	26	2.92 (0.883–10.6)	0.11*
TCS	2	7	16.8 (5.32–67.5)	13	16.2 (4.41–64.4)	0.31**
TCC*	0.2	87	0.02 (0.00–0.06)	88	0.01 (0.00–0.03)	0.35**
Abbreviations: BP, butyl paraben; BzP, benzyl paraben; 3,4-DHB, 3,4-dihydroxy benzoic acid; EP, ethyl paraben; 4-HB, 4-hydroxy benzoic acid; HP, heptyl paraben; IQR, interquartile range; LOQ, limit of quantification; MP, methyl paraben; PP, propyl paraben; OH-Et-P, ethyl-protocatechuic acid; OH-Me-P, methyl-protocatechuic acid; TCC, triclocarban; TCS, triclosan. ^***a***^LOQ for all urinary chemicals; limit of detection for creatinine. *p < 0.05. **p < 0.0001, Wilcoxon–Mann–Whitney test comparing median female and male creatinine-adjusted urinary chemical concentrations.						

When partners were modeled individually, no associations were observed between ln-transformed chemical concentrations and couple fecundity even after adjustment with the exception of the biomarker 4-HB (Table 3). Increasing concentrations of male partners’ urinary 4-HB was marginally associated with enhanced couple fecundity (FOR = 1.17; 95% CI: 1.00, 1.36). No associations were observed when modeling couples’ concentrations and fecundity (Table 3).

**Table 3:** Individual partners’ urinary antimicrobials and fecundability odds ratios (FORs), *n* = 501.

Chemical	Female partners			Male partners		
	Unadjusted FOR (95% CI)	Adjusted^*a*^ FOR (95% CI)	Adjusted^*b*^ FOR (95% CI)	Unadjusted FOR (95% CI)	Adjusted^*a*^ FOR (95% CI)	Adjusted^*b*^ FOR (95% CI)
MP	0.97 (0.87, 1.08)	0.96 (0.85, 1.09)	0.95 (0.83, 1.09)	1.00 (0.96, 1.04)	1.00 (0.96, 1.05)	1.01 (0.96, 1.05)
EP	1.01 (0.97, 1.06)	1.01 (0.97, 1.06)	1.02 (0.97, 1.06)	1.00 (0.97, 1.03)	1.00 (0.97, 1.03)	1.00 (0.96, 1.03)
PP	1.00 (0.97, 1.03)	1.00 (0.97, 1.03)	0.99 (0.96, 1.03)	1.01 (0.99, 1.02)	1.01 (0.99, 1.03)	1.01 (0.99, 1.03)
BP	1.01 (0.96, 1.06)	1.01 (0.95, 1.06)	1.00 (0.94, 1.07)	1.01 (0.98, 1.04)	1.01 (0.99, 1.05)	1.01 (0.98, 1.05)
BzP^*d*^	1.04 (0.70, 1.55)	0.98 (0.63, 1.52)	1.00 (0.67, 1.50)	1.20 (0.84, 1.70)	1.16 (0.81, 1.67)	1.20 (0.84, 1.71)
4-HB	1.08 (0.95, 1.22)	1.14 (0.96, 1.35)	1.10 (0.93, 1.32)	1.17 (1.00, 1.36)^*c*^	1.10 (0.90, 1.36)	1.07 (0.87, 1.33)
3,4-DHB	0.98 (0.85, 1.13)	0.99 (0.83, 1.18)	0.99 (0.82, 1.19)	0.98 (0.84, 1.14)	0.93 (0.78, 1.12)	0.94 (0.78, 1.15)
OH-MeP	0.98 (0.87, 1.10)	0.98 (0.87, 1.11)	0.98 (0.86, 1.11)	1.02 (0.92, 1.13)	1.01 (0.91, 1.13)	1.01 (0.90, 1.13)
OH-EtP	1.05 (0.95, 1.17)	1.04 (0.95, 1.14)	1.04 (0.95, 1.13)	1.01 (0.98, 1.05)	1.01 (0.98, 1.05)	1.01 (0.97, 1.05)
TCS	1.01 (0.96, 1.06)	1.01 (0.96, 1.06)	1.01 (0.95, 1.06)	1.01 (0.95, 1.07)	1.00 (0.95, 1.06)	1.01 (0.94, 1.07)
TCC^*d*^	0.91 (0.62, 1.33)	0.95 (0.63, 1.41)	0.89 (0.56, 1.42)	0.98 (0.69, 1.38)	1.00 (0.70, 1.43)	1.03 (0.68, 1.58)
Abbreviations: BP, butyl paraben; BzP, benzyl paraben; 3,4-DHB, 3,4-dihydroxy benzoic acid; EP, ethyl paraben; FOR, fecundability odds ratios; 4-HB, 4-hydroxy benzoic acid; MP, methyl paraben; OH-Et-P, ethyl-protocatechuic acid; OH-Me-P, methyl-protocatechuic acid; PP, propyl paraben; TCC, triclocarban; TCS, triclosan. ^***a***^Cox proportional odds models were adjusted for age, creatinine, BMI (25 ≤ BMI < 30, 30 ≤ BMI < 35, and ≥ 35 kg/m^2^ compared with BMI < 25 kg/m^2^), smoking status (cotinine dichotomized at a threshold of 10 ng/mL), race/ethnicity (dichotomized, nonwhite vs. white) and income (dichotomized at $70,000). ^***b***^Cox proportional odds models were adjusted for female age, difference between partners’ age, both partners’ creatinine, BMI (25 ≤ BMI < 30, 30 ≤ BMI < 35, and ≥ 35 kg/m^2^ compared with BMI < 25 kg/m^2^), smoking status (cotinine dichotomized at a threshold of 10 ng/mL), race/ethnicity (dichotomized, nonwhite vs. white), income (dichotomized at $70,000), and partner’s continuous concentrations of urinary chemicals. ^***c***^*p* < 0.05 before rounding. ^***d***^FOR and 95% CI reported are from models of dichotomous chemical concentrations (above/below LOQ).						

**Figure 1:**
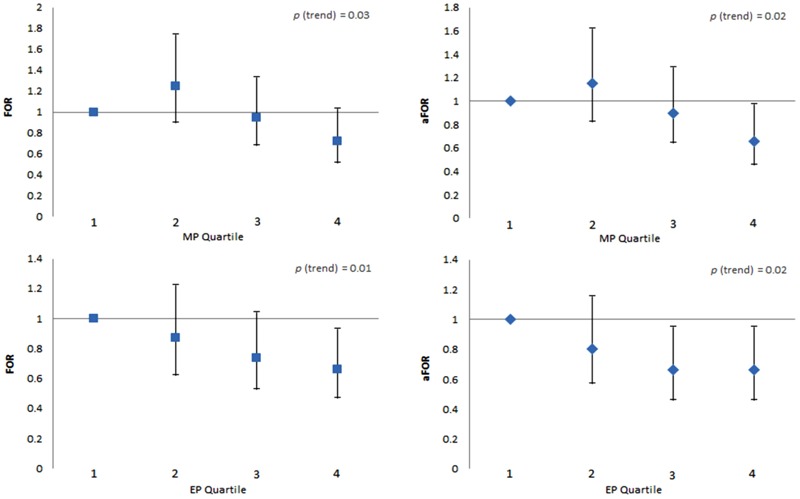
Female partners’ urinary MP and EP (in quartiles) and fecundability odds ratios. Abbreviations: EP, ethyl paraben; FOR, fecundability odds ratio; MP, methyl paraben. Error bars represent 95% confidence intervals. FORs are from unadjusted Cox proportional odds models. aFORs were adjusted for age, creatinine, BMI (25 ≤ BMI < 30, 30 ≤ BMI < 35, and ≥ 35 kg/m^2^ compared with BMI < 25 kg/m^2^), smoking status (cotinine dichotomized at a threshold of 10 ng/mL), race/ethnicity (dichotomized, nonwhite vs. white), and income (dichotomized at $70,000).

Figure 1 presents the unadjusted and adjusted FORs for chemicals (in quartiles) found having a significant association with couple fecundity when modeling each partner of the couple. Females in the 4th quartile (≥ 104 ng/mL) of MP concentrations had a 28% reduction in fecundity that increased to a 34% reduction in adjusted models (FOR = 0.72; 95% CI: 0.51, 1.03, and aFOR = 0.66; 95% CI: 0.45, 0.97, respectively) when compared with women in the 1st quartile (< 12 ng/mL). Moreover, a significant (*p* = 0.02) trend was observed. A similar relationship was observed when comparing females in the highest (≥ 5.62 ng/mL) versus lowest quartile (< 0.27 ng/mL) of EP in both unadjusted and adjusted models (FOR = 0.66; 95% CI: 0.47, 0.93, and aFOR = 0.66; 95% CI: 0.46, 0.95, respectively), reflecting in a significant trend (*p* = 0.02). No association was observed between the remaining parabens and couple fecundity when modeled in quartiles (see Table S2). With regard to male partners’ concentrations, significant FORs were observed for BP and 4-HB but only in the unadjusted analysis (Figure 1). No significant associations were observed for any of the remaining parabens, as quantified in men (see Table S2).

**Figure 2:**
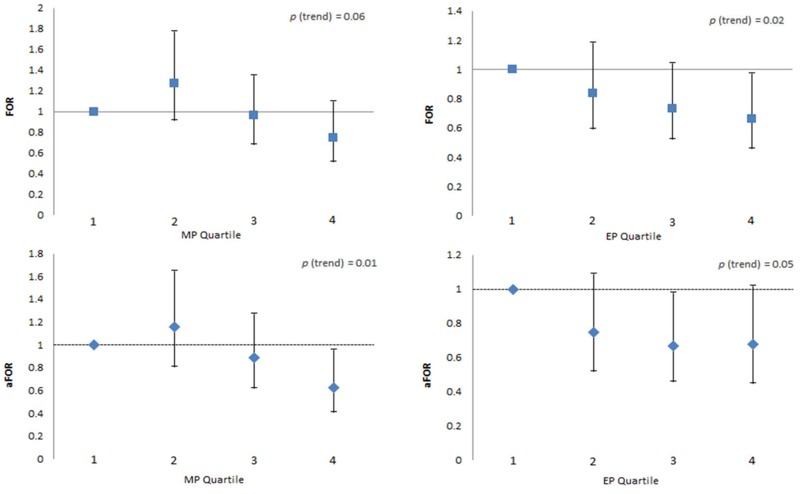
Female partners’ urinary MP and EP and fecundability odds ratios (couple-based analysis). Abbreviations: EP, ethyl paraben; FOR, fecundability odds ratio; MP, methyl paraben. Error bars represent 95% confidence intervals. FORs are from unadjusted Cox proportional odds models of both female and male partners’ urinary chemical concentrations. aFORs are from Cox proportional odds models adjusted for female age, difference between partners’ age, both partners’ creatinine, BMI (25 ≤ BMI < 30, 30 ≤ BMI < 35, and ≥ 35 kg/m^2^ compared with BMI < 25 kg/m^2^), smoking status (cotinine dichotomized at a threshold of 10 ng/mL), race/ethnicity (dichotomized, nonwhite vs. white), income (dichotomized at $70,000), and urinary chemical concentrations.

When both partners’ concentrations were modeled given the low correlations between their concentrations (ranging from *r* = –0.07 to 0.10; Table 2), female partners’ MP and EP concentrations remained significantly associated with diminished fecundity by 37% (aFOR = 0.63; 95% CI: 0.41, 0.96) and 33% (aFOR = 0.67; 95% CI: 0.46, 0.98), respectively, after adjusting for the male partners’ concentrations (Figure 2). Findings not achieving significance in the couple based model are presented in Table S3.

## Discussion

In the first prospective assessment of couples’ fecundity in the context of preconception urinary antimicrobial chemicals, we found little evidence that parabens, TCS, and TCC were associated with couple fecundity within models of ln-transformed chemical concentrations. However, a 34% reduction in couple fecundity was observed for female partners with urinary MP concentrations in the highest quartile and urinary EP concentrations in the third quartile, compared with the MP concentrations < 12 ng/mL and EP concentrations < 0.27 ng/mL, after adjustment. Likewise, we observed a 37% and 33% reduction in couple fecundity for female MP and EP concentrations when accounting for both partners’ urinary chemical concentration in covariate-adjusted analyses. In the LIFE Study, median antimicrobial concentrations were approximately 10 times higher for female than for male partners. Median urinary MP concentrations for females (60.0 μg/g creatinine) and males (6.37 μg/g creatinine) in the LIFE Study were generally lower than those reported in the NHANES (National Health and Nutrition Examination Survey) cross-sectional survey (147 μg/g and 21.1 μg/g creatinine, respectively) (Calafat et al. 2008). These findings suggest that for couple fecundity to be negatively associated with preconception urinary MP and EP, the female partner’s concentration needs to be at the higher end of the distribution.

Comparison of our findings with previous work is limited considering that only a few epidemiological studies have focused on nonpersistent environmental chemicals as they relate to human fecundity (Vélez et al. 2015a, 2015b), and even fewer have included both partners despite TTP being a couple-dependent outcome (Buck Louis et al. 2014a, 2014c; Specht et al. 2015). We are unaware of any previous work on preconception exposure to antimicrobial chemicals (i.e., parabens, TCS) in relation to couple fecundity measured as TTP, precluding a more complete interpretation of our findings. Nonetheless, though not directly comparable, in a previous study assessing TCS and fecundity as measured by retrospectively reported TTP in pregnant women, a 14% reduction in fecundity was reported among women in the highest (> 72 ng/mL) versus lowest quartile of TCS (Vélez et al. 2015a); a finding that our analysis did not corroborate.

Still, our results are strengthened by several components of the LIFE cohort study design as well as analytic methods. Primarily the prospective assessment of TTP is a sensitive measure of couple fecundity. Women in LIFE were screened for pregnancy at study enrollment with the use of the Clearblue® Digital home pregnancy test, capable of detecting pregnancy with 99% accuracy (false positive results range, 0–0.3%, depending on pregnancy test lot) when used from the day of expected menstrual cycle (Tomlinson et al. 2008). Moreover, the use of digital pregnancy tests removes ambiguity in the interpretation of test results based on color and symbol. Women without a positive hCG test at enrollment in the study were eligible to participate in the cohort, ensuring the accurate capture of prospectively assessed TTP. Additionally, predictors of estimated antimicrobial exposure were thoughtfully considered in the present analysis. Potential chemical associations with couple fecundity were explored with ln-transformed urinary concentrations and then by categorizing urinary chemical concentrations to assess potential linear and nonlinear relationships. Also, we explored models of unadjusted chemical concentrations to avoid the potential bias induced by using creatinine-adjusted chemical concentrations in studies of human health (Cocker et al. 2011; Weaver et al. 2016); all models were also performed on nonimputed data resulting in marginally significant findings that did not remain in the imputation analysis. Furthermore, our models included the novel quantification of hydroxylated metabolites of MP (OH-MeP) and EP (OH-EtP), and nonspecific paraben biomarkers 4-HB and 3,4-DHB (Wang and Kannan 2013) as predictors of estimated exposures in relation to couple fecundity. Of note is the observation that the measured concentrations of these metabolites were much higher than those of the parent parabens, highlighting the stability of such compounds and usefulness as exposure biomarkers (Wang and Kannan 2013). Despite the use of such novel biomarkers, our findings are limited by reliance on a single spot urine sample collected at enrollment, given the short biologic half-lives of parabens, TCS, and TCC (Sandborgh-Englund et al. 2006; Soni et al. 2005). Still, serial measurements of parabens and TCS over a period of several months have been reported to have relatively high correlation reflecting continual exposure: ICCs ranging from 0.40 to 0.65 in nonpregnant adult females and males of reproductive age from U.S. (Massachusetts), Belgian, and Danish populations (Dewalque et al. 2015; Lassen et al. 2013). Therefore, because 90% of pregnancies in the LIFE Study occurred within the first six menstrual cycles and 38% within cycles 0–1 (Buck Louis et al. 2012), the potential for exposure misclassification may be reduced but not eliminated. We attempted to evaluate this consideration by restricting our analysis to couples with a TTP < 2 cycles (*n* = 167) and continued to observe a diminished fecundity for the women in the highest vs. lowest quartile of MP (0.59; 95% CI: 0.20, 1.70). Furthermore, our findings were generally robust when we restricted our analysis to only include those couples with measured chemical concentrations.

Despite the paucity of data in the context of biologic plausibility, given the robust, significant associations observed between female MP and EP and couple fecundity in our cohort, potential mechanisms have been considered. Primarily, the estrogenic activity of parabens has largely been established in animal studies (Boberg et al. 2010; Darbre and Harvey 2008; Karpuzoglu et al. 2013). Although animal studies have demonstrated MP and EP to be less estrogenic than PP and BP (Vo et al. 2010; Witorsch and Thomas 2010), we observed significant associations between higher female concentrations of MP and EP in urine and couple fecundity. Furthermore, the observed relationship between female urinary concentrations of MP and EP and couple fecundity in our analysis may, in part, be explained by other mechanisms. Oxidative stress is a purported factor of female reproductive disorders including endometriosis and polycystic ovary syndrome, which have implications for reduced fecundity (Agarwal et al. 2012; Ruder et al. 2008). In general, parabens have been correlated with the urinary biomarker of oxidative stress, 8-hydroxy-2´-deoxyguanosine, in humans (Asimakopoulos et al. 2016), whereas MP and EP have are suspected of reacting with oxygen in the skin to produce a free radical or reactive oxygenated species (Nishizawa et al. 2006). Also, higher concentrations of MP were measured in female than in male urine samples in the LIFE Study, which may reflect a greater use of personal care products among female partners (Manová et al. 2013; Wu et al. 2010), and may therefore partly explain our urinary MP and couple fecundity findings. However, we are also aware of the potential for chance findings, having performed multiple comparisons in the current analysis.

The lack of association between antimicrobial concentrations in males and couple fecundity may be explained by the relatively low urinary concentrations of parabens, TCS, and TCC measured among males in our cohort. Therefore we were unable to corroborate the findings of a previous *in vitro* assessment that demonstrated anti-androgenic effects of several parabens and TCS in response to large micromolar concentrations with the use of a cell-based human androgen receptor–mediated bioassay (Chen et al. 2007). Additionally we recognize that our models did not adjust for semen quality. Although semen quality is an important factor of couple fecundity, to avoid model overadjustment (Schisterman et al. 2009) we decided against the adjustment for semen quality in the present analysis given the lack of an association between any of the 35 semen quality parameters and TTP in our study cohort in a previous analysis (Buck Louis et al. 2014b).

In light of the observational nature of this work, the absence of longitudinal chemical measurements and residual confounding, cautious interpretation of our findings is warranted. Our results await corroboration by larger prospective cohort studies of repeated preconception urinary measures of parabens and other antimicrobial chemicals in the context of couple fecundity.

## Conclusion

Female but not male partners’ preconception urinary concentration of MP and EP were associated with a 37% and 33% reduction in couple fecundity, as measured by a longer TTP.

## Supplemental Material

(160 KB) PDF
