# Supplementary material for: Urinary Concentrations of Parabens and Other Antimicrobial Chemicals and Their Association with Couples’ Fecundity
Source: Environ Health Perspect. 2016 Jun 10;125(4):730–6. doi: 10.1289/EHP189 (PMC5381974; doi:10.1289/EHP189)
Supplement: (160 KB) PDF [file EHP189.s001.acco.pdf]

Environ Health Perspect
DOI: 10.1289/EHP189

Note to readers with disabilities: *EHP* strives to ensure that all journal content is accessible to all readers. However, some figures and Supplemental Material published in *EHP* articles may not conform to 508 standards due to the complexity of the information being presented. If you need assistance accessing journal content, please contact ehp508@niehs.nih.gov. Our staff will work with you to assess and meet your accessibility needs within 3 working days.

Supplemental Material

Urinary Concentrations of Parabens and Other Antimicrobial Chemicals and Their Association with Couples' Fecundity

Melissa M. Smarr, Rajeshwari Sundaram, Masato Honda, Kurunthachalam Kannan, and Germaine M. Buck Louis

Table of Contents

Table S1. Unadjusted preconception antimicrobial phenolic urinary concentrations by partner, LIFE Study, 2005-2009

Table S2. Unadjusted and adjusted fecundability odds ratios and 95% confidence intervals for preconception urinary concentrations of parabens and other antimicrobial chemicals modeled as quartiles of biomarkers of exposure in partner-specific models of estimated exposure, LIFE Study, 2005-2009

Table S3. Unadjusted and adjusted fecundability odds ratios and 95% confidence intervals for preconception urinary concentrations of parabens and other antimicrobial chemicals modeled as quartiles of biomarkers of exposure in couple-based models of estimated exposure, LIFE Study, 2005-2009

Table S1. Unadjusted preconception antimicrobial phenolic urinary concentrations by partner, LIFE Study, 2005-2009.

Chemical (ng/mL)	All<br>**Median (IQR)**	Females<br>**Median (IQR)**	Males<br>**Median (IQR)**
Parabens			
MP **	17.2 (4.03 ,67.1)	31.9 (12.0, 104)	6.55 (2.13, 26.4)
EP **	0.56 (0.21, 3.02)	1.09 (0.27, 5.62)	0.37 (0.17, 1.26)
PP **	4.63 (0.89, 21.3)	12.1 (3.54, 35.6)	1.45 (0.49, 5.55)
BP **	0.12 (0.02,1.21)	0.59 (0.08, 2.84)	0.03 (0.01, 0.17)
BzP *	0.02 (0.00, 0.04)	0.02 (0.00, 0.04)	0.02 (0.00, 0.04)
HP	0.00 (0.00, 0.00)	0.00 (0.00, 0.00)	0.00 (0.00, 0.00)
Paraben Metabolites			
4-HB**	551 (300, 916)	493 (269, 895)	614 (348, 920)
3,4-DHB**	33.0 (18.4, 57.3)	33.0 (18.2, 58.1)	33.0 (18.6, 57.2)
OH-MeP**	21.2 (11.5, 44.9)	21.5 (10.7, 43.7)	20.9 (12.3, 45.6)
OH-EtP**	3.92 (1.23, 12.9)	4.40 (1.41, 13.5)	3.57 (1.17, 11.8)
Antibacterials			
TCS	14.8 (4.02, 60.2)	11.7 (3.55, 54.2)	17.8 (4.42, 77.1)
TCC *	0.01 (0.00, 0.03)	0.01 (0.00, 0.03)	0.01 (0.00, 0.03)

Abbreviations: IQR, interquartile range; MP, methyl paraben; EP, ethyl paraben; PP, propyl paraben; BP, butyl paraben; BzP, benzyl paraben; HP, heptyl paraben; 4-HB, 4-hydroxy benzoic acid; 3,4-DHB, 3,4-dihydroxy benzoic; OH-Me-P, methyl-protocatechuic acid; OH-Et-P, ethyl-protocatechuic acid; TCS, triclosan; TCC, triclocarban.

Table S2. Unadjusted and adjusted fecundability odds ratios and 95% confidence intervals for preconception urinary concentrations of parabens and other antimicrobial chemicals modeled as quartiles of biomarkers of exposure in partner-specific models of estimated exposure, LIFE Study, 2005-2009[a]

	2nd Quartile	3rd Quartile	4th Quartile	
**Chemical**	**FOR (95% CI)**	**FOR (95% CI)**	**FOR (95% CI)**	**p-trend**
Unadjusted Models				
Females				
MP	1.25 (0.90, 1.74)	0.95 (0.68, 1.33)	0.72 (0.51, 1.03)	0.03
EP	0.87 (0.62, 1.22)	0.74 (0.53, 1.04)	0.66 (0.47, 0.93)	0.01
PP	1.07 (0.77, 1.49)	0.88 (0.63, 1.24)	0.82 (0.59, 1.16)	0.16
BP	0.91 (0.65, 1.27)	0.99 (0.70, 1.39)	0.77 (0.54, 1.10)	0.21
4-HB	1.06 (0.75, 1.49)	1.11 (0.78, 1.57)	1.17 (0.83, 1.64)	0.35
3,4-DHB	0.96 (0.68, 1.34)	1.19 (0.84, 1.67)	0.84 (0.59, 1.20)	0.56
OH-MeP	0.94 (0.67, 1.33)	0.96 (0.69, 1.35)	0.93 (0.66, 1.30)	0.70
OH-EtP	0.87 (0.62, 1.23)	0.89 (0.63, 1.25)	1.23 (0.87, 1.73)	0.23
TCS	1.02 (0.72, 1.43)	0.84 (0.59, 1.20)	0.92 (0.65, 1.30)	0.44
Males				
MP	1.27 (0.90, 1.81)	0.91 (0.63, 1.30)	0.92 (0.64, 1.33)	0.31
EP	1.22 (0.87, 1.72)	1.10 (0.78, 1.56)	0.87 (0.61, 1.23)	0.33
PP	1.00 (0.70, 1.42)	0.95 (0.68, 1.34)	0.86 (0.62, 1.19)	0.33
BP	1.26 (0.89, 1.79)	1.45 (1.02, 2.05)	1.22 (0.85, 1.76)	0.23
4-HB	1.04 (0.74, 1.46)	1.54 (1.08, 2.18)	1.47 (1.04, 2.08)	0.01
3,4-DHB	1.12 (0.78, 1.60)	0.96 (0.66, 1.40)	0.91 (0.63, 1.31)	0.45
OH-MeP	1.10 (0.79, 1.55)	0.81 (0.57, 1.15)	0.98 (0.70, 1.39)	0.55
OH-EtP	1.10 (0.76, 1.58)	1.14 (0.79, 1.66)	1.16 (0.81, 1.66)	0.39
TCS	1.02 (0.71, 1.47)	0.99 (0.69, 1.41)	1.08 (0.75, 1.56)	1.02
Adjusted Models[b]				
Females				
MP	1.15 (0.82, 1.62)	0.90 (0.64, 1.29)	0.66 (0.45, 0.97)	0.02
EP	0.80 (0.57, 1.15)	0.66 (0.46, 0.95)	0.66 (0.46, 0.95)	0.02
PP	0.97 (0.70, 1.36)	0.82 (0.58, 1.16)	0.76 (0.52, 1.11)	0.10
BP	1.00 (0.71, 1.41)	0.97 (0.69, 1.37)	0.81 (0.57, 1.16)	0.25
4-HB	1.22 (0.83, 1.78)	1.27 (0.85, 1.91)	1.36 (0.88, 2.10)	0.18
3,4-DHB	1.00 (0.70, 1.42)	1.14 (0.78, 1.66)	0.88 (0.58, 1.34)	0.70
OH-MeP	0.96 (0.67, 1.39)	0.96 (0.67, 1.37)	0.94 (0.66, 1.34)	0.72
OH-EtP	0.82 (0.58, 1.18)	0.82 (0.57, 1.17)	1.11 (0.78, 1.59)	0.53
TCS	0.94 (0.66, 1.35)	0.83 (0.57, 1.22)	0.86 (0.59, 1.25)	0.35
Males				
MP	1.22 (0.85, 1.74)	0.91 (0.62, 1.34)	0.92 (0.62, 1.36)	0.39
EP	1.11 (0.78, 1.57)	0.98 (0.69, 1.40)	0.84 (0.58, 1.21)	0.26
PP	0.98 (0.68, 1.40)	1.01 (0.71, 1.46)	0.84 (0.59, 1.19)	0.36
BP	1.24 (0.86, 1.78)	1.42 (0.99, 2.04)	1.15 (0.78, 1.71)	0.41
4-HB	1.03 (0.70, 1.53)	1.42 (0.92, 2.19)	1.34 (0.86, 2.09)	0.11

3,4-DHB	1.15 (0.79, 1.67)	0.90 (0.60, 1.34)	0.84 (0.55, 1.29)	0.28
OH-MeP	1.20 (0.85, 1.70)	0.79 (0.55, 1.15)	0.97 (0.68, 1.39)	0.40
OH-EtP	1.08 (0.74, 1.58)	1.15 (0.77, 1.70)	1.06 (0.72, 1.55)	0.72
TCS	0.97 (0.66, 1.41)	0.95 (0.65, 1.38)	1.02 (0.69, 1.51)	0.95

Abbreviations: FOR, Fecundability odds ratios; MP, methyl paraben; EP, ethyl paraben; PP, propyl paraben; BP, butyl paraben; 4-HB, 4-hydroxy benzoic acid; 3,4-DHB, 3,4-dihydroxy benzoic; OH-Me-P, methyl-protocatechuic acid; OH-Et-P, ethyl-protocatechuic acid; TCS, triclosan.

[a] In all models, the 1st quartile is the reference for quartile comparisons.

[b]Cox proportional odds models were adjusted for age, creatinine, BMI ($25 \leq BMI < 30$, $30 \leq BMI < 35$, and ≥ 35 kg/m^2 compared with BMI < 25 kg/ m^2), smoking status (cotinine dichotomized at a threshold of 10 ng/mL), race/ethnicity (dichotomized, Non-White vs. White) and income (dichotomized at $70,000).

Table S3. Unadjusted and adjusted fecundability odds ratios and 95% confidence intervals for preconception urinary concentrations of parabens and other antimicrobial chemicals modeled as quartiles of biomarkers of exposure in couple-based models of estimated exposure, LIFE Study, 2005-2009[a].

Chemical	2nd Quartile FOR (95% CI)	3rd Quartile FOR (95% CI)	4th Quartile FOR (95% CI)	p-trend
Unadjusted Models				
Females				
MP	1.27 (0.91, 1.77)	0.96 (0.68, 1.35)	0.75 (0.51, 1.10)	0.06
EP	0.84 (0.59, 1.18)	0.73 (0.52, 1.04)	0.67 (0.46, 0.97)	0.02
PP	1.09 (0.78, 1.53)	0.91 (0.64, 1.29)	0.85 (0.59, 1.22)	0.24
BP	0.88 (0.63, 1.24)	0.93 (0.65, 1.32)	0.71 (0.48, 1.04)	0.11
4-HB	1.02 (0.72, 1.45)	1.02 (0.71, 1.46)	1.06 (0.75, 1.50)	0.73
3,4-DHB	0.96 (0.68, 1.36)	1.19 (0.84, 1.68)	0.86 (0.60, 1.23)	0.62
OH-MeP	0.95 (0.67, 1.35)	0.99 (0.70, 1.39)	0.95 (0.67, 1.34)	0.80
OH-EtP	0.86 (0.61, 1.21)	0.87 (0.61, 1.23)	1.21 (0.84, 1.74)	0.33
TCS	1.00 (0.71, 1.42)	0.81 (0.56, 1.18)	0.87 (0.59, 1.28)	0.31
Males				
MP	1.30 (0.91, 1.85)	0.96 (0.67, 1.39)	0.98 (0.67, 1.44)	0.65
EP	1.26 (0.89, 1.78)	1.21 (0.84, 1.72)	0.99 (0.68, 1.45)	0.94
PP	0.99 (0.69, 1.42)	0.97 (0.68, 1.38)	0.89 (0.62, 1.26)	0.54
BP	1.25 (0.88, 1.77)	1.53 (1.06, 2.19)	1.31 (0.89, 1.94)	0.13
4-HB	1.03 (0.73, 1.46)	1.53 (1.07, 2.19)	1.45 (1.02, 2.07)	0.01
3,4-DHB	1.12 (0.78, 1.61)	0.96 (0.66, 1.41)	0.94 (0.64, 1.36)	0.49
OH-MeP	1.10 (0.79, 1.55)	0.81 (0.57, 1.16)	0.99 (0.70, 1.41)	0.60
OH-EtP	1.10 (0.76, 1.59)	1.16 (0.79, 1.70)	1.08 (0.74, 1.58)	0.58
TCS	1.06 (0.73, 1.55)	1.05 (0.71, 1.53)	1.18 (0.78, 1.78)	0.46
Adjusted Models[b]				
Females				
MP	1.16 (0.81, 1.65)	0.89 (0.62, 1.27)	0.63 (0.41, 0.96)	0.01
EP	0.75 (0.52, 1.09)	0.67 (0.46, 0.98)	0.68 (0.45, 1.02)	0.05
PP	0.99 (0.70, 1.40)	0.83 (0.57, 1.19)	0.72 (0.48, 1.07)	0.06
BP	0.96 (0.68, 1.37)	0.88 (0.61, 1.26)	0.74 (0.50, 1.10)	0.13
4-HB	1.16 (0.79, 1.72)	1.17 (0.77, 1.78)	1.18 (0.76, 1.83)	0.46
3,4-DHB	0.99 (0.67, 1.45)	1.15 (0.77, 1.72)	0.88 (0.57, 1.35)	0.68
OH-MeP	0.93 (0.65, 1.34)	0.94 (0.65, 1.36)	0.96 (0.66, 1.39)	0.78
OH-EtP	0.78 (0.54, 1.13)	0.76 (0.53, 1.10)	1.09 (0.74, 1.60)	0.71
TCS	0.95 (0.66, 1.38)	0.82 (0.54, 1.23)	0.83 (0.53, 1.30)	0.33
Males				
MP	1.27 (0.88, 1.83)	0.94 (0.63, 1.40)	1.02 (0.67, 1.57)	0.81
EP	1.13 (0.78, 1.63)	1.02 (0.70, 1.49)	0.92 (0.61, 1.39)	0.59
PP	1.04 (0.71, 1.50)	1.08 (0.75, 1.56)	0.91 (0.62, 1.33)	0.7
BP	1.22 (0.84, 1.78)	1.47 (0.99, 2.17)	1.22 (0.80, 1.85)	0.31
4-HB	0.99 (0.66, 1.47)	1.34 (0.85, 2.11)	1.26 (0.80, 1.99)	0.18
3,4-DHB	1.16 (0.79, 1.70)	0.91 (0.60, 1.37)	0.89 (0.58, 1.38)	0.38

OH-MeP	1.20 (0.83, 1.72)	0.78 (0.53, 1.14)	0.96 (0.66, 1.39)	0.41
OH-EtP	1.07 (0.71, 1.60)	1.18 (0.77, 1.81)	1.00 (0.66, 1.51)	0.84
TCS	1.12 (0.74, 1.69)	1.04 (0.68, 1.58)	1.21 (0.76, 1.92)	0.49

Abbreviations: FOR, Fecundability odds ratios; MP, methyl paraben; EP, ethyl paraben; PP, propyl paraben; BP, butyl paraben; 4-HB, 4-hydroxy benzoic acid; 3,4-DHB, 3,4-dihydroxy benzoic; OH-Me-P, methyl-protocatechuic acid; OH-Et-P, ethyl-protocatechuic acid; TCS, triclosan.

[a] In all models, the 1st quartile is the reference for quartile comparisons.

[b] Cox proportional odds models were adjusted for female age, difference between partners' age, both partner's: creatinine, BMI ($25 \leq$ BMI < 30, $30 \leq$ BMI < 35, and ≥ 35 kg/m^2 compared with BMI < 25 kg/ m^2), smoking status (cotinine dichotomized at a threshold of 10 ng/mL), race/ethnicity (dichotomized, Non-White vs. White), income (dichotomized at $70,000) and partner's continuous concentrations of urinary biomarkers.
